# Supplementary material for: Towards the Development of Long Circulating Phosphatidylserine (PS)- and Phosphatidylglycerol (PG)-Enriched Anti-Inflammatory Liposomes: Is PEGylation Effective?
Source: Pharmaceutics. 2021 Feb 19;13(2):282. doi: 10.3390/pharmaceutics13020282 (PMC7922817; doi:10.3390/pharmaceutics13020282)
Supplement: Supplementary file 1 [file pharmaceutics-13-00282-s001.pdf]

pharmaceutics

MDPI

Supplementary Materials

Towards the Development of Long Circulating Phosphatidylserine (PS) and Phosphatidylglycerol (PG) Enriched Anti-Inflammatory Liposomes: Is PEGylation Effective?

Miriam E. Klein, Max Rieckmann, Daniel Sedding, Gerd Hause, Annette Meister, Karsten Mäder, Henrike Lucas

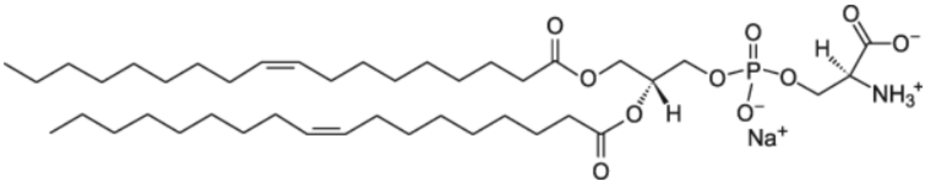

(A)

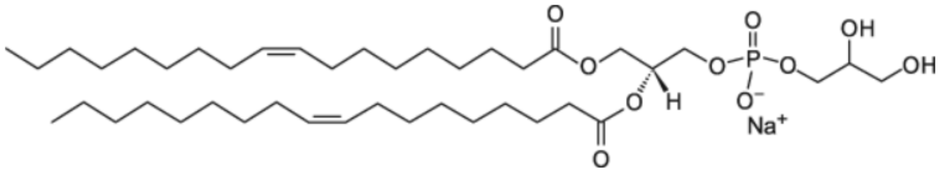

(B)

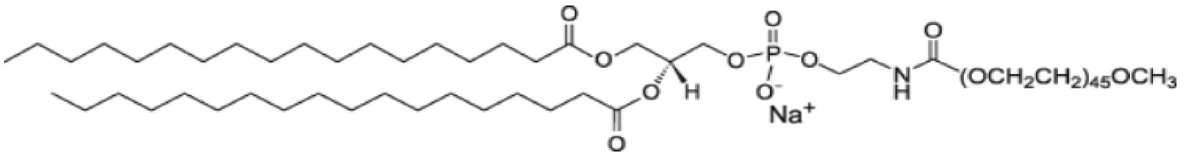

(C)

Figure S1. The chemical structures of DOPS, DOPG and PEG are presented. S100 is a natural product derived from soybean. A chemical structure is not provided. It consists mainly of phospholipids with palmitoyl, stearyl, oleoyl, linoleoyl or linoenoyl fatty acid chains. **(A)** 1,2-dioleoyl-*sn*-glycero-3-phospho-L-serine sodium (DOPS). **(B)** 1,2-dioleoyl-sn-glycero-3-phospho-1'-rac-glycerol sodium (DOPG). **(C)** 1,2-distearoyl-sn-glycero-3-phosphoethanolamine-N-[methoxy(polyethylenglycerol)-2000] sodium (MPEG-2000-DSPE, PEG).

Table S1. Plate 1—organ positions for *ex vivo* fluorescence imaging via IVIS FI system.

	**1**	**2**	**3**	**4**
A	Blood	Liver	Spleen	Duodenum
B	Heart	Kidney	Pancreas	Caecum
C	Lung	Bladder	Gut	Colon

Table S2. Plate 2—organ positions for ex vivo fluorescence imaging via IVIS FI system.

	**1**	**2**	**3**	**4**
A	Brain	Femur	Preputial	
B	Skin	Muscle	Testis	
C	Adipose tissue	Uterus / Ovaries	Vesicular gland	

Table S3. Experimental parameters for the IVIS Spectrum fluorescence imaging system.

**Parameter**	**Value**
Excitation (DiR)	745 nm (30 nm band with)
Emission (DiR)	800 nm (20 nm band with)
Field of view:	
Mouse	D (FOV 22.5 cm)
Tissue	C (FOV 13.0 cm)
Exposure parameter:	Automatic mode
Exposure time	0.5–60 s
Binning	1–8
F/Stop	1–8
n_{max} of photons	6000

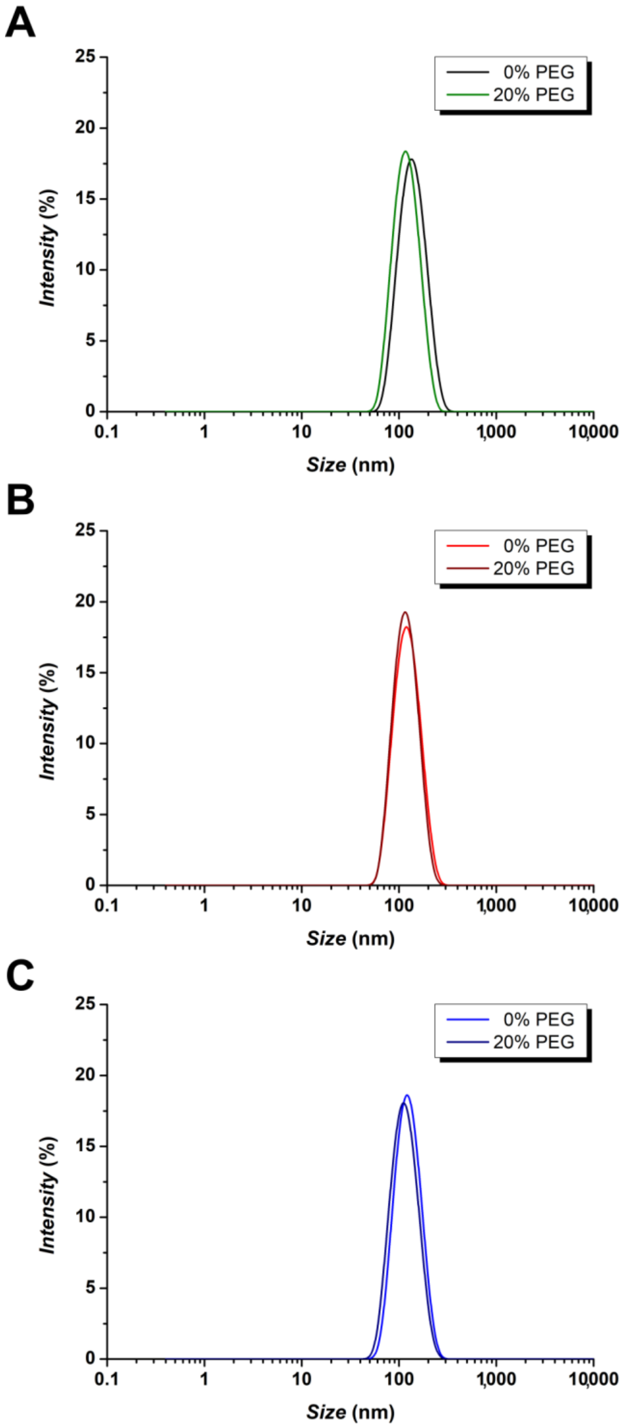

Figure S2. DLS histograms of representative measurements of different phospholipid nanodispersions, **(A)** S100-liposomes (conventional liposomes = 0% PEG, 'stealth' liposomes = 20% PEG), **(B)** DOPS-liposomes (conventional liposomes = 0% PEG, 'stealth' liposomes = 20% PEG), **(C)** DOPG-liposomes (conventional liposomes = 0% PEG, 'stealth' liposomes = 20% PEG).

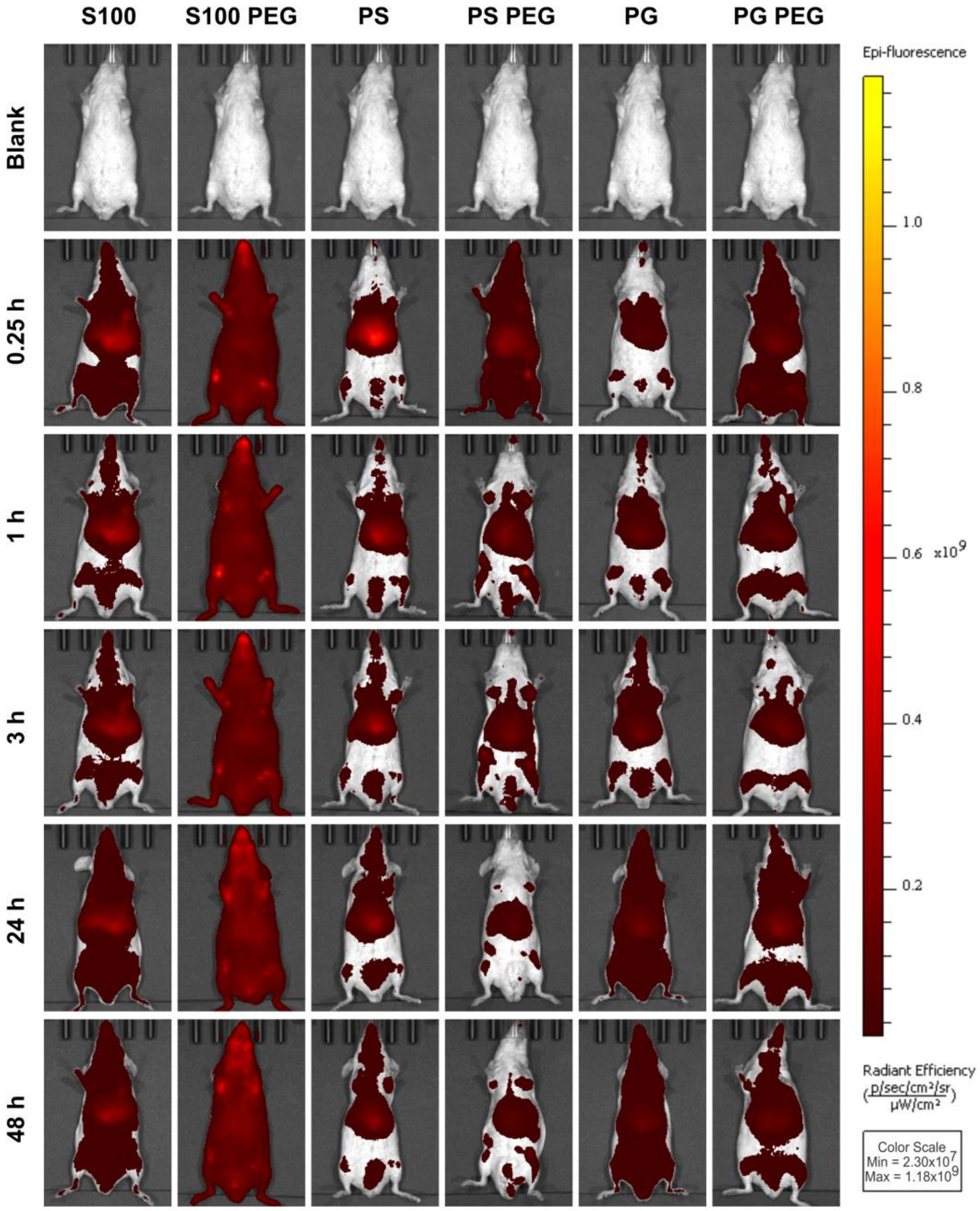

Figure S3. In vivo liposome pharmacokinetics differed in distribution and abdominal accumulation, depending on both formulation and PEGylation. DiR-loaded liposome-treated male mice (ventral perspective), as assessed by in vivo FI. The total radiant efficiency (TRE) of representative mice of each group and time points are shown. For direct comparison, radiant efficiency was normalized.

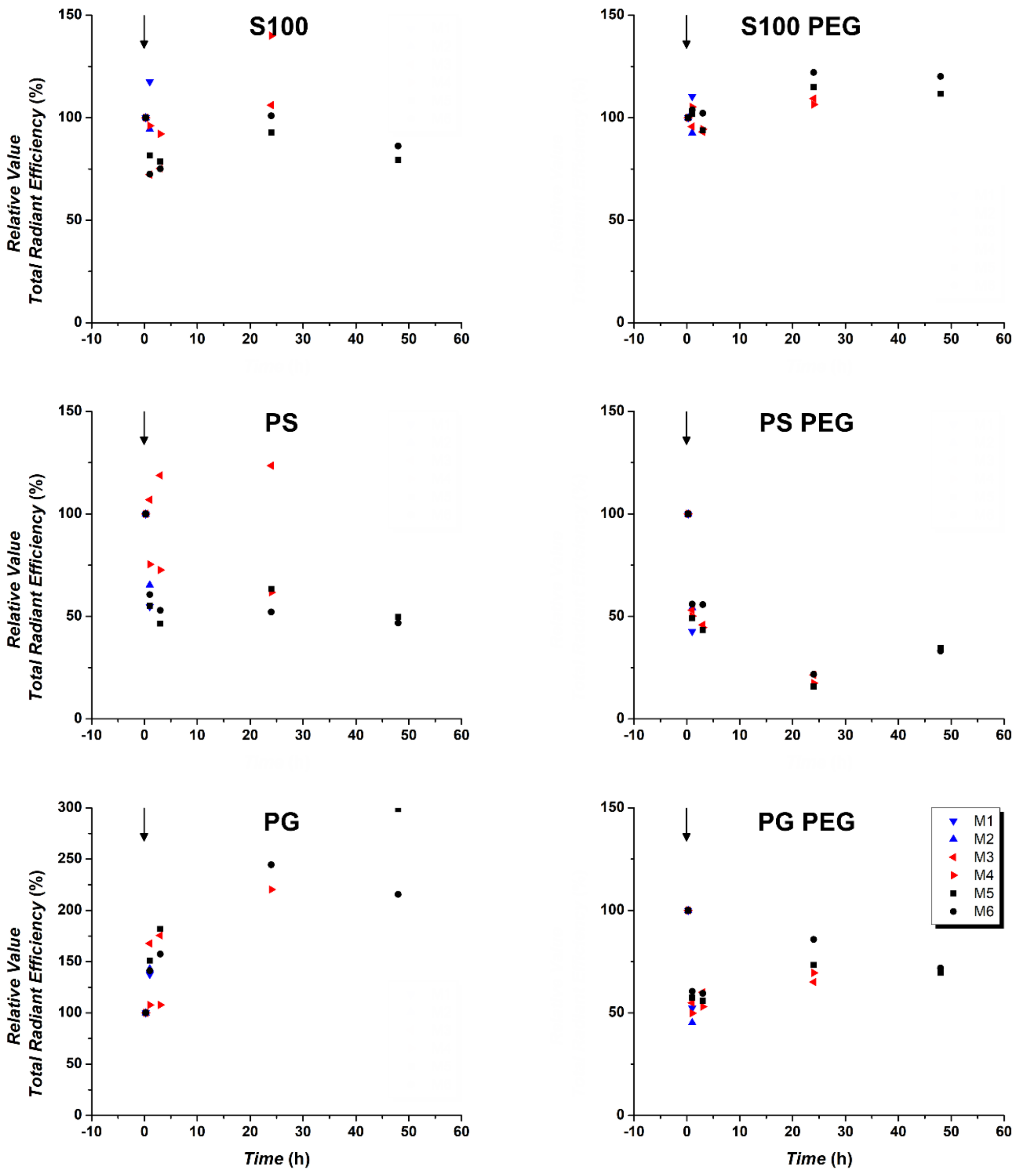

Figure S4. Quantification of in vivo fluorescence signal showed prolonged circulation of S100 PEG and fast clearance of other formulations. Signals were evaluated from defined ROI in DiR-loaded liposome-treated male mice (ventral perspective), as assessed by in vivo FI at 0.25, 1, 3, 24 and 48 h post injectionem. M1 to M6 represent individual mouse values. M1 and M2 were sacrificed at t = 1 h, M3 and M4 at t = 24 h and M5 and M6 at t = 48 h. Presented values are relative to t = 0.25 h.

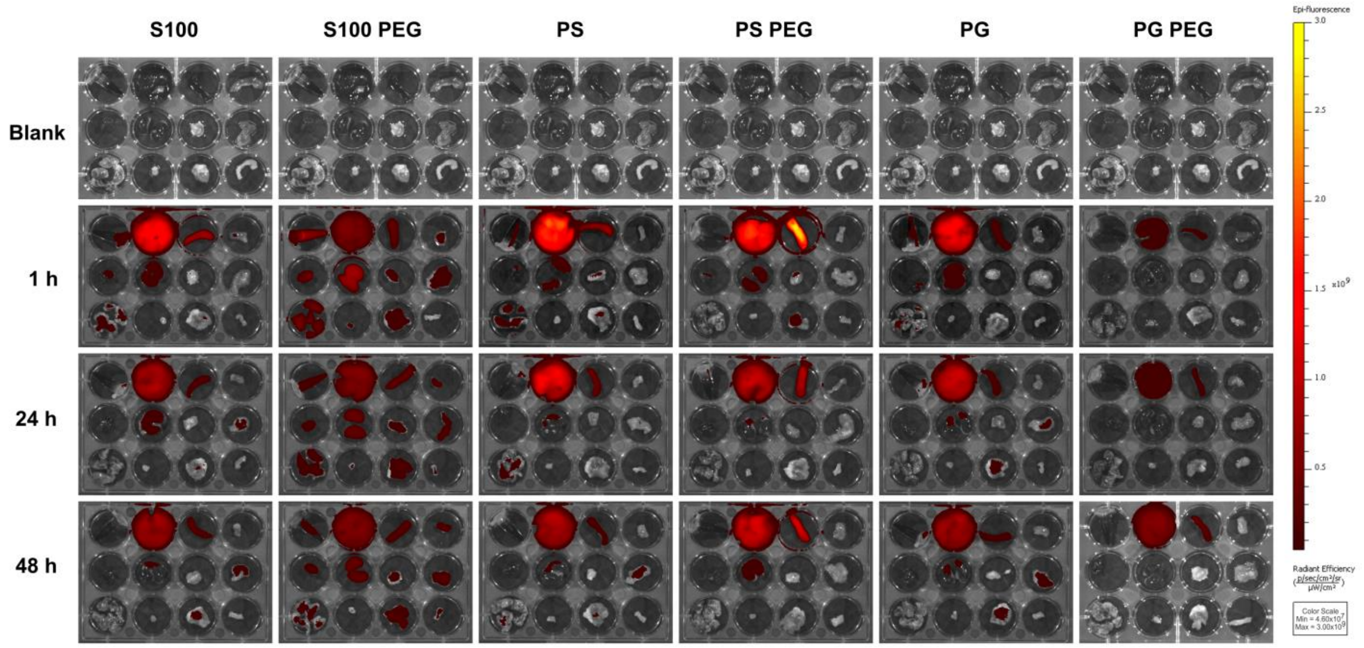

Figure S5. Ex vivo biodistribution of DiR-loaded liposomal formulations (Plate 1) in female mice, assessed by FI. The total radiant efficiency (TRE) of representative mice of each group are shown. For direct comparison, radiant efficiency was normalized.

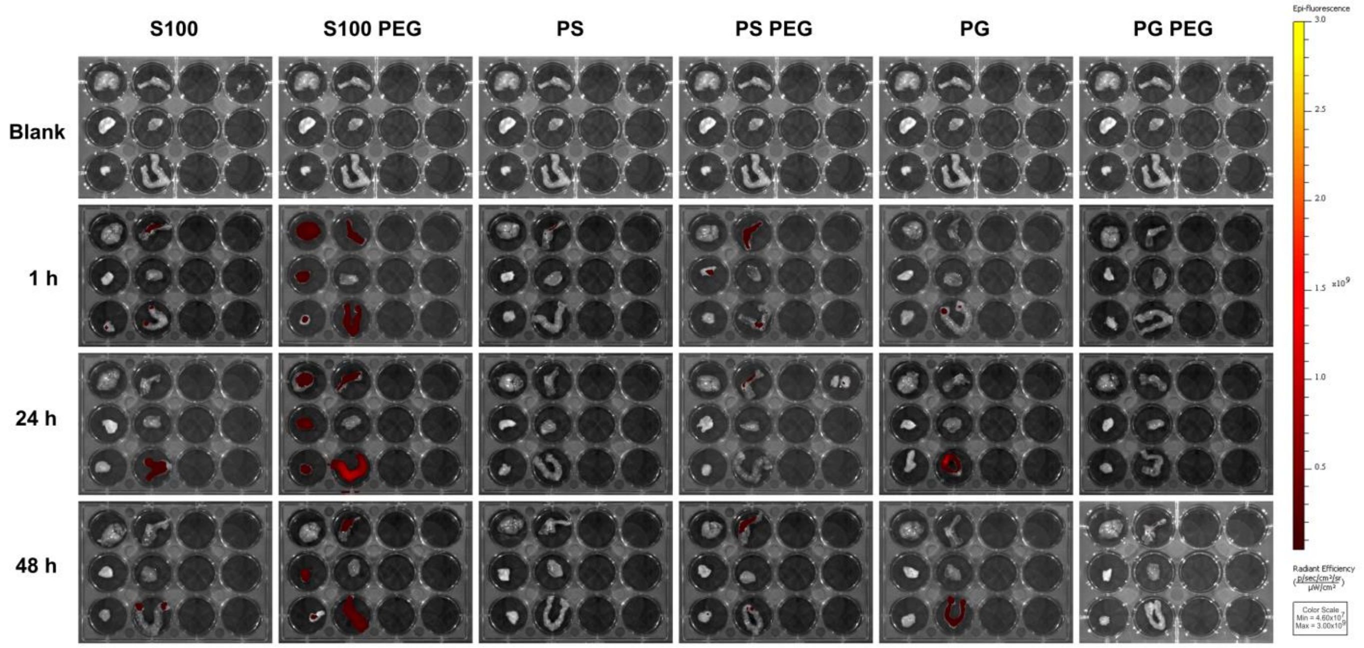

Figure S6. Ex vivo biodistribution of DiR-loaded liposomal formulations (Plate 2) in female mice, assessed by FI. The total radiant efficiency (TRE) of representative mice of each group are shown. For direct comparison, radiant efficiency was normalized.

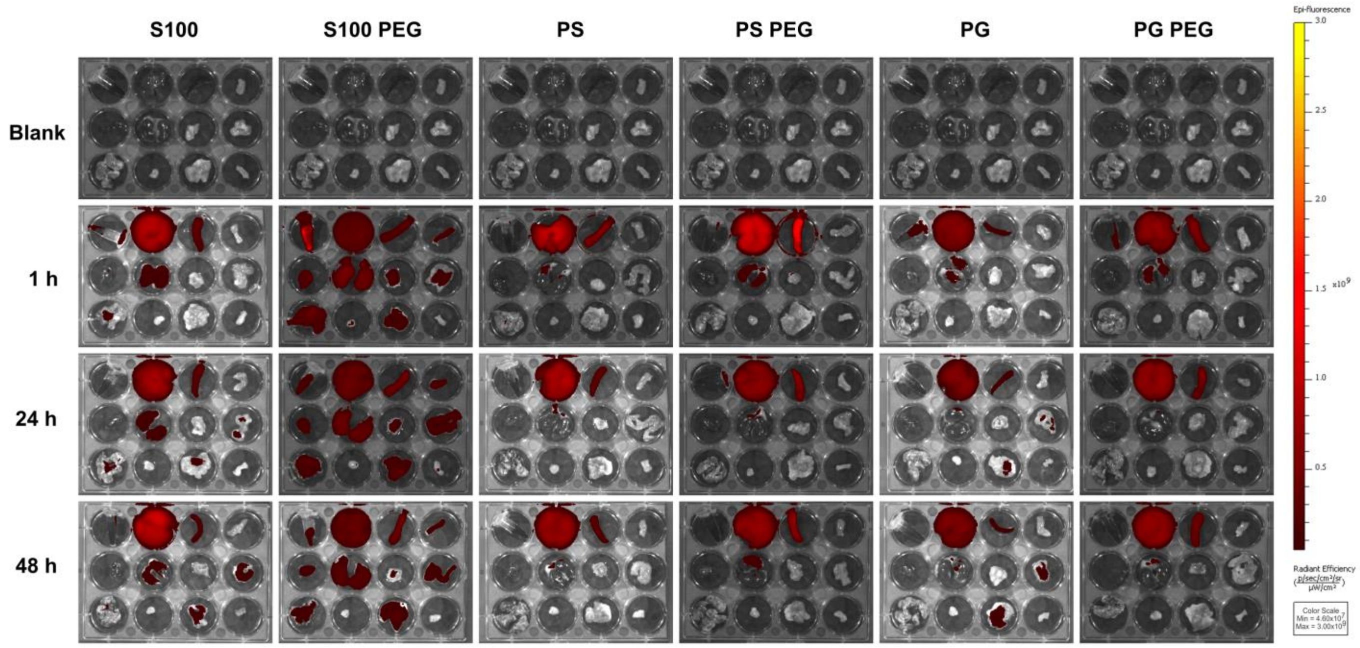

Figure S7. Ex vivo biodistribution of DiR-loaded liposomal formulations (Plate 1) in male mice, assessed by FI. The total radiant efficiency (TRE) of representative mice of each group are shown. For direct comparison, radiant efficiency was normalized.

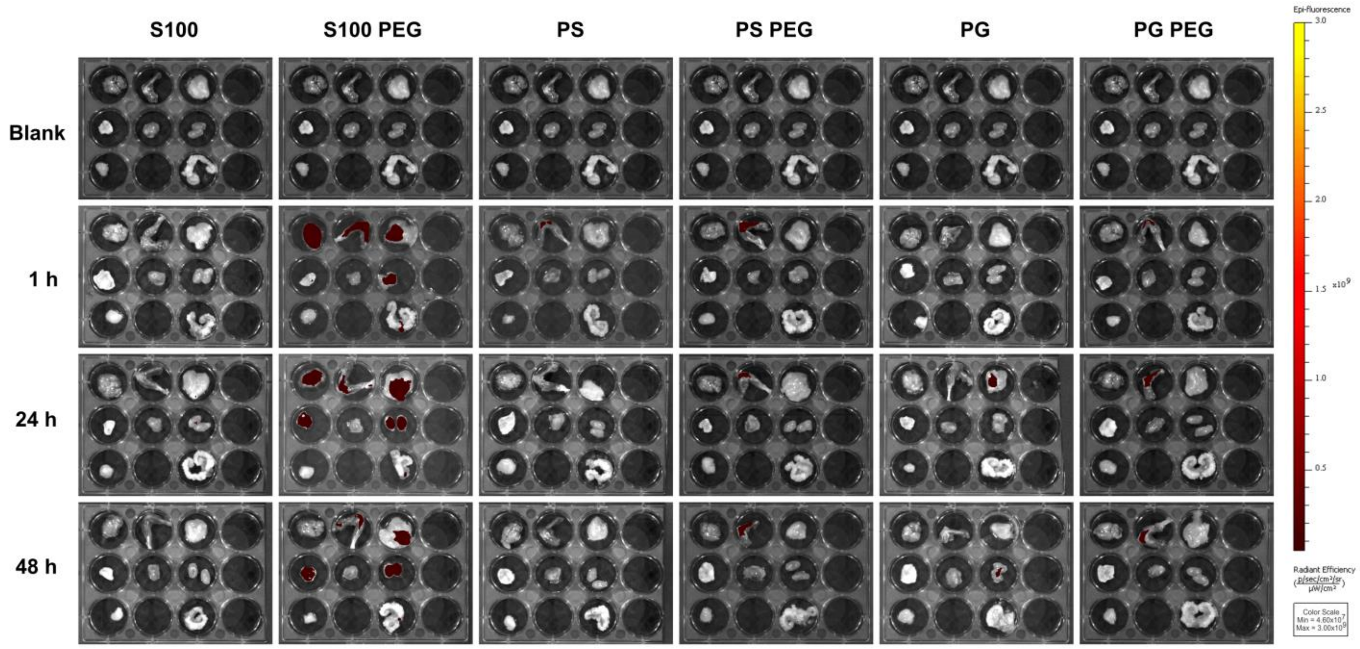

Figure S8. Ex vivo biodistribution of DiR-loaded liposomal formulations (Plate 2) in male mice, as assessed by FI. The total radiant efficiency (TRE) of representative mice of each group are shown. For direct comparison, radiant efficiency was normalized.

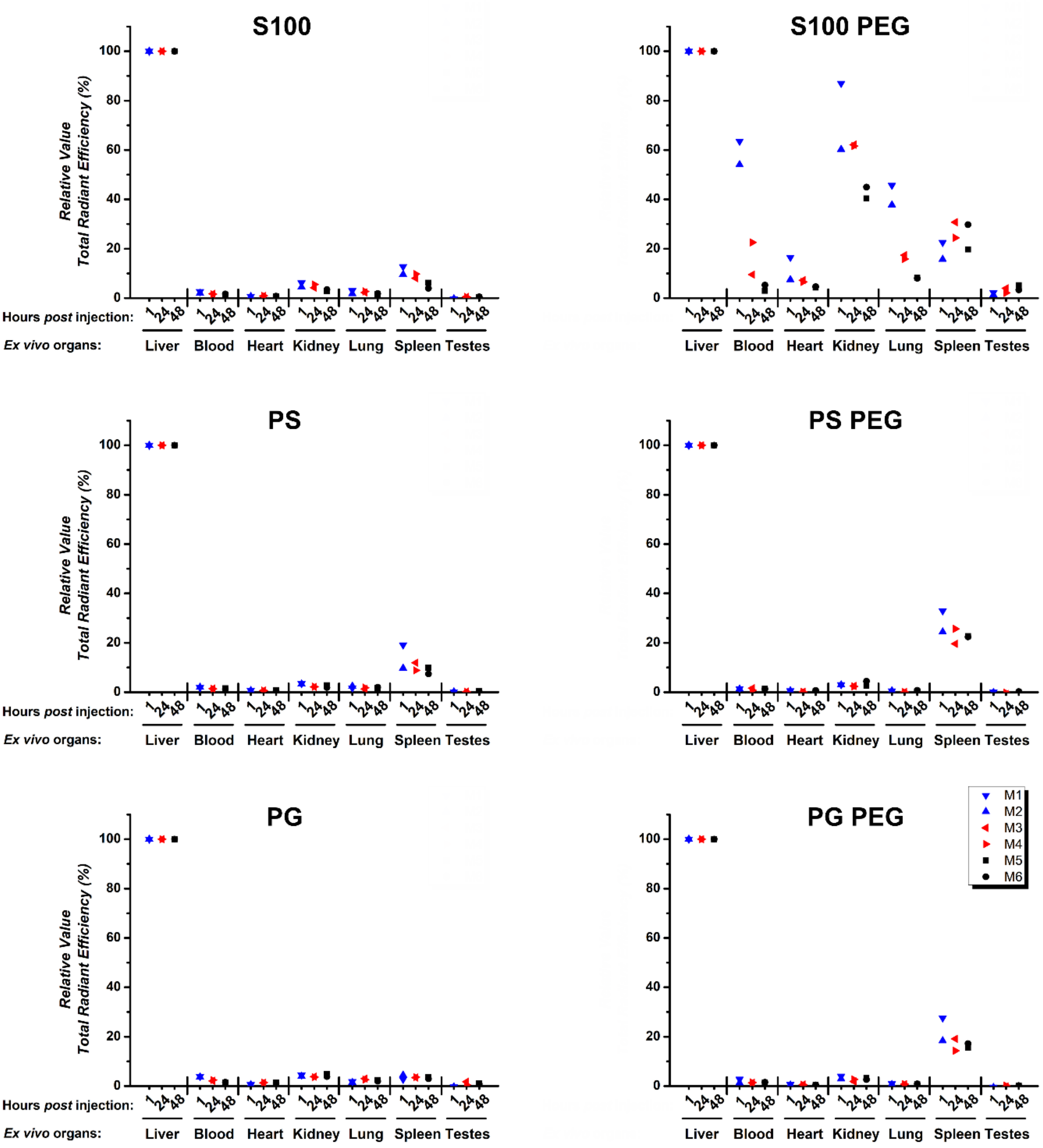

Figure S9. Quantification of ex vivo single organ total radiant efficiencies (TRE) (preselected) shows systemic and spatial accumulation of S100 PEG and fast clearance of other formulations via liver and spleen. Signals were evaluated from defined ROI in DiR-loaded liposome-treated excised single organs, as assessed by ex vivo FI. M1 to M6 represent individual male mouse values. M1 and M2 were sacrificed at t = 1 h, M3 and M4 at t = 24 h and M5 and M6 at t = 48 h. Presented values are relative to the TRE of the respective liver.

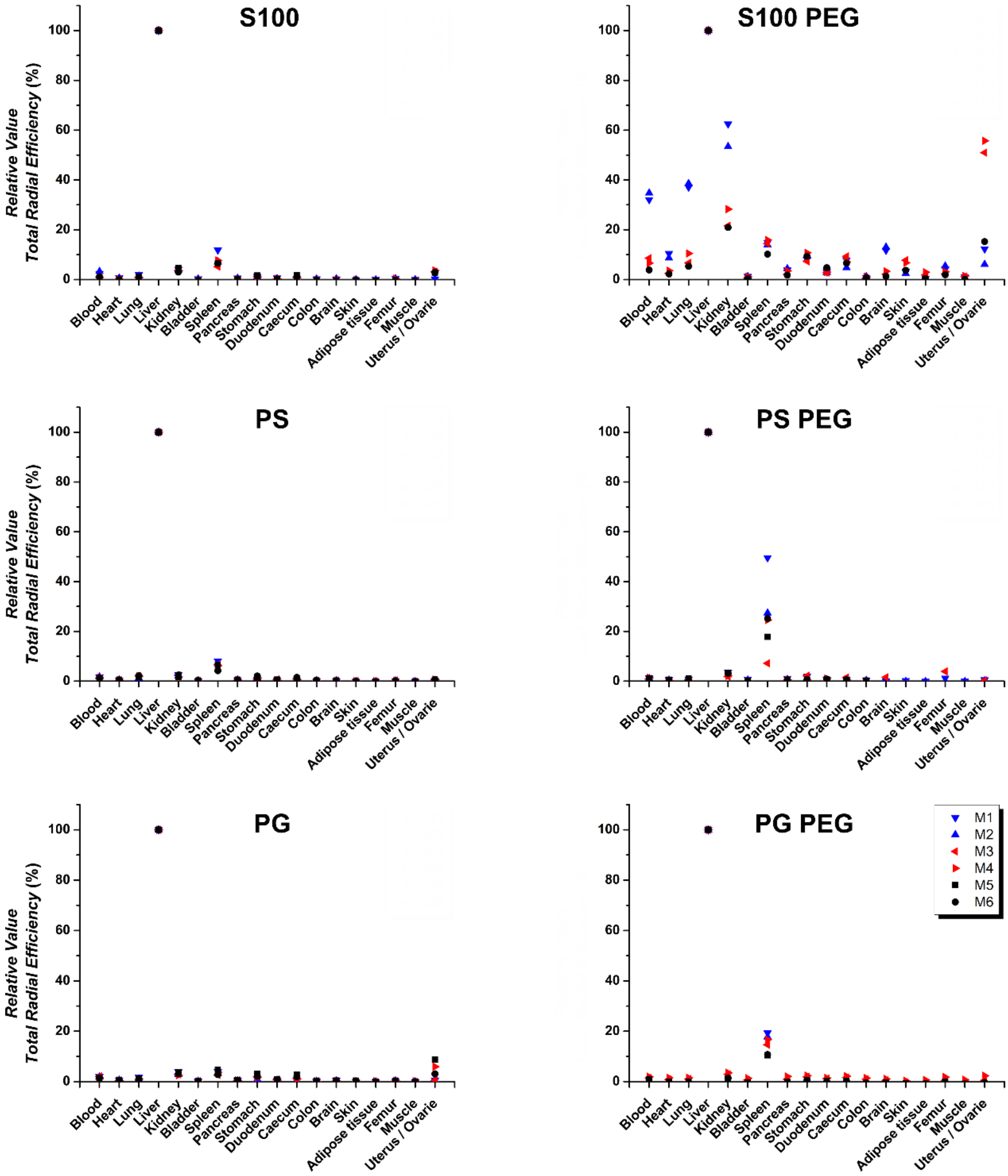

Figure S10. Quantification of ex vivo single organ total radiant efficiencies (TRE) shows systemic accumulation of S100 PEG and fast clearance of other formulations via liver and spleen. Signals were evaluated from defined ROI in DiR-loaded liposome-treated excised single organs, as assessed by ex vivo FI. M1 to M6 represent individual female mouse values. M1 and M2 were sacrificed at t = 1 h, M3 and M4 at t = 24 h and M5 and M6 at t = 48 h. Presented values are relative to the TRE of the respective liver.

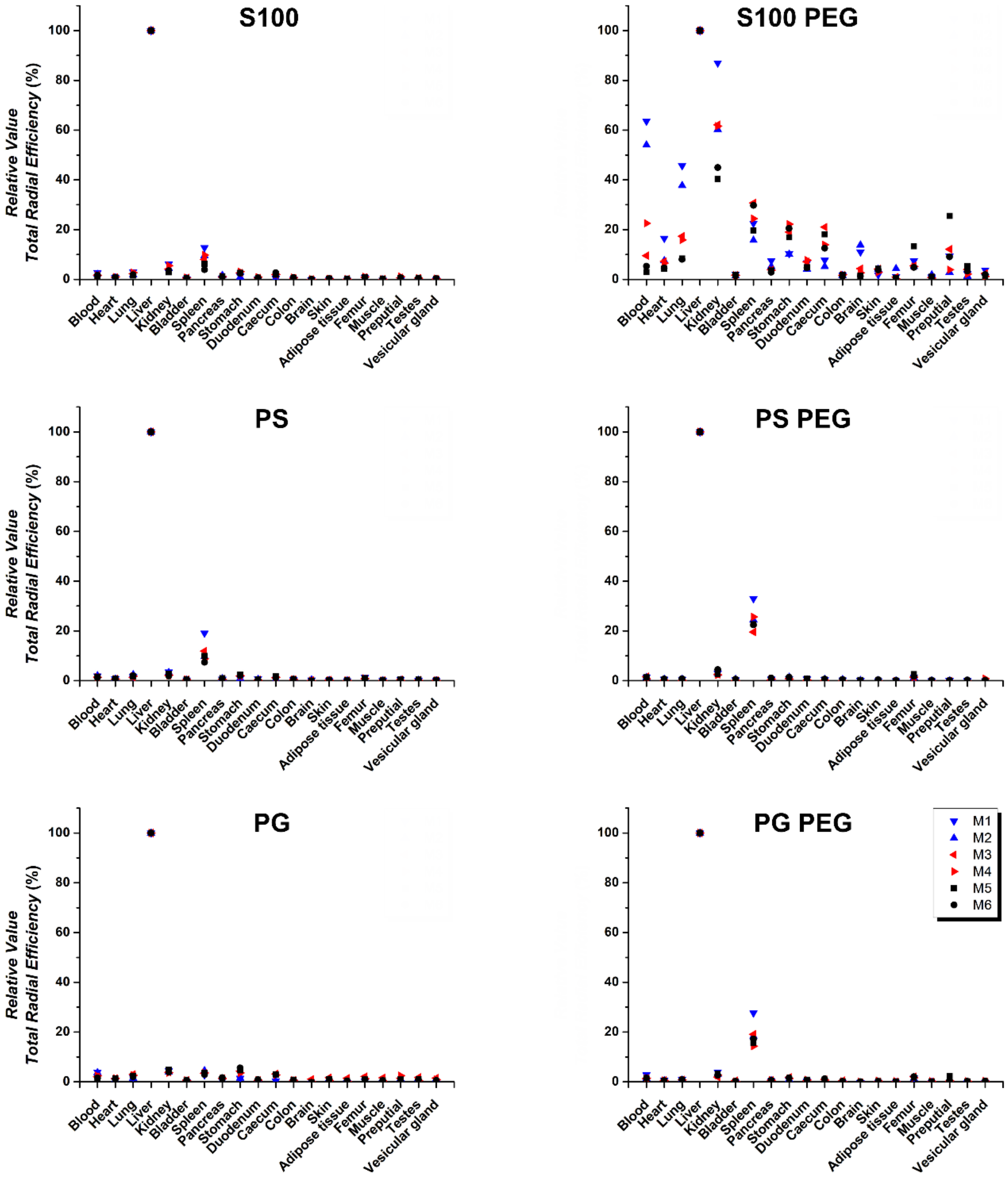

Figure S11. Quantification of ex vivo single organ total radiant efficiencies (TRE) shows systemic accumulation of S100 PEG and fast clearence of other formulations via liver and spleen. Signals were evaluated from defined ROI in DiR-loaded liposome-treated excised single organs, as assessed by ex vivo FI. M1 to M6 represent individual male mouse values. M1 and M2 were sacrificed at t = 1 h, M3 and M4 at t = 24 h and M5 and M6 at t = 48 h. Presented values are relative to the TRE of the respective liver.

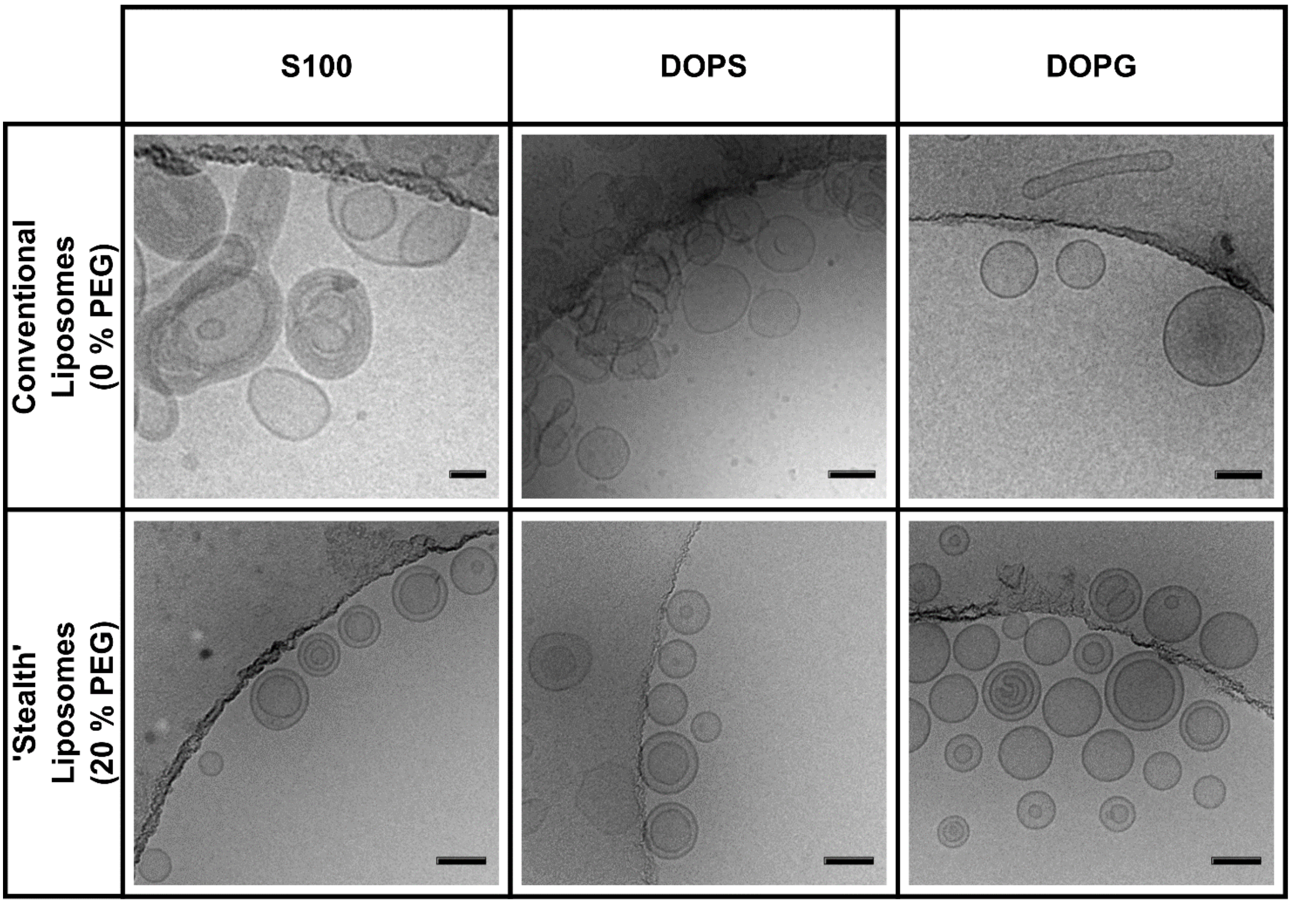

Figure S12. Cryo-TEM images of PS and PG enriched nanodispersions. Scale bar represents 100 nm.
